# Long Noncoding RNA PVT1 as a Novel Diagnostic Biomarker and Therapeutic Target for Melanoma

**DOI:** 10.1155/2017/7038579

**Published:** 2017-02-07

**Authors:** Xiangjun Chen, Guozhen Gao, Sha Liu, Li Yu, Dexiong Yan, Xingwei Yao, Weijing Sun, Dezhi Han, Hao Dong

**Affiliations:** ^1^Department of Burn and Plastic Surgery, The 253rd Hospital of PLA, Hohhot, Inner Mongolia 010051, China; ^2^Intensive Care Unit, The 253rd Hospital of PLA, Hohhot, Inner Mongolia 010051, China; ^3^Department of General Surgery, The 253rd Hospital of PLA, Hohhot, Inner Mongolia 010051, China

## Abstract

Accumulating evidences indicated that plasmacytoma variant translocation 1 (PVT1) plays vital roles in several cancers. However, the expression, functions, and clinical values of PVT1 in melanoma are still unknown. In this study we measured the expression of PVT1 in clinical tissues and serum samples and explored the diagnostic value of PVT1 for melanoma and the effects of PVT1 on melanoma cell proliferation, cell cycle, and migration. Our results, combined with publicly available PVT1 expression data, revealed that PVT1 is upregulated in melanoma tissues compared with nonneoplastic nevi tissues. Serum PVT1 level is significantly increased in melanoma patients compared with age and gender-matched nonmelanoma controls with melanocytic nevus. Receiver operating characteristic curve analyses revealed that serum PVT1 level could sensitively discriminate melanoma patients from controls. Furthermore, serum PVT1 level indicted melanoma dynamics. Functional experiments showed that overexpression of PVT1 promotes melanoma cells proliferation, cell cycle progression, and migration, while depletion of PVT1 significantly inhibits melanoma cells proliferation, cell cycle progression, and migration. Collectively, our results indicate that PVT1 functions as an oncogene in melanoma and could be a potential diagnostic biomarker and therapeutic target for melanoma.

## 1. Introduction

The incidence of melanoma is increasing quickly worldwide for the past 30 years and will continue to increase in the future [1]. It is predicted that melanoma will be an enormous public healthy and economic burden for human [2]. Currently, there are 160,000 estimated new cases of melanoma and 48,000 estimated deaths from melanoma in the world every year [3]. Although early stage melanoma could be cured by surgery section, the treatment for later stage melanoma is still difficult and less efficient [4]. Therefore, it is critical to diagnose melanoma at early stage and develop more efficient molecular targeted therapies for melanoma [5]. To be disappointed, there are still no broadly used serum biomarkers for melanoma early diagnosis.

Long noncoding RNA (lncRNA) is a novel class of RNA transcripts longer than 200 nucleotides and with limited protein-coding potential [6, 7]. Many reports have shown that lncRNAs have critically important roles in various biological processes and are dysregulated in many diseases, particularly in cancers [8–11]. Furthermore, multiple reports have revealed that several lncRNAs are detectable in serum and function as sensitive biomarkers for early diagnosis of cancers, such as H19 for gastric cancer [12] and XIST and HIF1A-AS1 for nonsmall cell lung cancer [13]. The lncRNA plasmacytoma variant translocation 1 (PVT1) is well known for its critical roles in carcinogenesis and progression of many cancers, including hepatocellular carcinoma [14], lung cancer [15], and breast cancer [16]. Moreover, increasing evidences indicate that PVT1 functions in a cell-type and tissues-specific manner [17]. However, the roles and clinical values of PVT1 in melanoma are still unknown.

In this study, we focused on the expression, functions, and diagnostic values of PVT1 in melanoma. We detected PVT1 expression in melanoma tissues in both public database and our own cohort. We also measured PVT1 expression in the serum of melanoma patients and analyzed its diagnostic values for melanoma. Furthermore, we explored the functions of PVT1 in melanoma cell proliferation, cell cycle, and migration.

## 2. Materials and Methods

### 2.1. Patients and Samples

A total of 51 melanoma patients and 47 age and gender-matched nonmelanoma controls with melanocytic nevus were enrolled into this study at the 253rd Hospital of PLA (Hohhot, Inner Mongolia, China). All the participants were confirmed by histological diagnosis. Venus blood was collected from fasting participants and centrifuged at 3000 rpm for 15 min at 4°C. The supernatant was immediately collected and stored at −80°C until use. Thirty malignant melanoma tissues and twenty skin tissues with melanocytic nevus were obtained from these participants undergoing surgery section at the 253rd Hospital of PLA. All tissue samples were immediately frozen in liquid nitrogen and stored at −80°C until use after surgery. The Review Board of the 253rd Hospital of PLA approved this study, and all the participants signed written informed consent in accordance with the Declaration of Helsinki.

### 2.2. Cell Cultures

The human melanoma cell line A375 was purchased from Cell Resource Center, Shanghai Institutes for Biological Sciences (Shanghai, China). A375 cells were cultured in DMEM medium supplemented with 10% fetal bovine serum (Gibco, CA, USA) in 5% CO_2_ atmosphere at 37°C.

### 2.3. RNA Extraction and Quantitative PCR (qPCR)

Total RNA was isolated from serum, tissues, or cells using Trizol Reagent (Invitrogen, CA, USA) following the manufacturer's manual. Reverse transcription was carried out using PrimeScript™ II 1st Strand cDNA Synthesis Kit (Takara, Dalian, China) following the manufacturer's manual. qPCR was subsequently carried out using SYBR® Premix Ex Taq™ II (Takara) on ABI StepOne Plus system (Applied Biosystems, CA, USA) following the manufacturer's manual. The gene expression was calculated using 2^−ΔΔCt^ method. GAPDH was used as endogenous control. The primers sequences are as follows: PVT1 (NR_003367), 5′-ATAGATCCTGCCCTGTTTGC-3′ (forward) and 5′-CATTTCCTGCTGCCGTTTTC-3′ (reverse); PVT1-1, 5′-GAATGTGAACAATGGGAACC-3′ (forward) and 5′-GCAGCAACAGGAGAAGCAA-3′ (reverse); PVT1-2, 5′-ACAGAGAAGATGAAGAGATG-3′ (forward) and 5′-GAAAGTTAGAAAACAGTGGG-3′ (reverse); PVT1-3, 5′-GACTACAGCTGGAAGACAG-3′ (forward) and 5′-GGCTCAGAAAATACTTGAAC-3′ (reverse); PVT1-5, 5′-CGAGTAGCTGGGACTACA-3′ (forward) and 5′-GCTGACAATCCTTGAAAAG-3′ (reverse); GAPDH, 5′-GGAGCGAGATCCCTCCAAAAT-3′ (forward) and 5′-GGCTGTTGTCATACTTCTCATGG-3′ (reverse).

### 2.4. Vectors Construction and Transfection

The full-length PVT1 (NR_003367) transcript was PCR amplified from cDNA derived from A375 cell with the Ex Taq® Hot Start Version DNA Polymerase (Takara) and subcloned into the* Kpn* I and* Bam*H I sites of pcDNA3.1(+) plasmid (Invitrogen). The primers sequences are as follows: 5′-GGGGTACCCTCCGGGCAGAGCGCGTGTG-3′ (forward) and 5′-CGGGATCCTAGACACGAGGCCGGCCACGC-3′ (reverse). To inhibit PVT1 expression, two oligonucleotides for shRNAs were synthesized and inserted into the shRNA expression vector pGPH1/Neo (GenePharma, Shanghai, China). The shRNAs sequences are as follows: shRNA #1, 5′-GCTTCAACCCATTACGATTTC-3′; shRNA #2, 5′-GGACTTGAGAACTGTCCTTAC-3′. A scrambled shRNA was used as negative control. Vectors were transfected into melanoma cells using Lipofectamine 3000 (Invitrogen) following the manufacturer's manual.

### 2.5. Generation of Melanoma Cells Stably Overexpressing or Depleting PVT1

To obtain PVT1 stably overexpressed melanoma cells, A375 cells were transfected with pcDNA3.1-PVT1 vectors and selected with neomycin for four weeks. To obtain PVT1 stably depleted melanoma cells, A375 cells were transfected with PVT1 shRNAs expression plasmids and selected with neomycin for four weeks.

### 2.6. Cell Proliferation Assays

Cell counting kit-8 (CCK-8) assays and ethynyl deoxyuridine (EdU) incorporation assays were performed to assess cells proliferation. For CCK-8 assays, a total of approximately 5.0 × 10^3^ cells/well was plated in 96-well plate. After culture for 24, 48, and 72 hours, cell viability was measured by the cell counting kit-8 (Dojindo Laboratories, Kumamoto, Japan) and a microplate reader following the manufacturer's manual. EdU incorporation assays were performed using an EdU Kit (Roche, Mannheim, Germany) following the manufacturer's manual. Representative images were acquired by Zeiss Axiophot Photomicroscope (Carl Zeiss, Oberkochen, Germany) and the results were quantified by Image-Pro plus 6.0 software.

### 2.7. Cell Cycle Analyses

Cell cycle distribution was measured using the Cell Cycle Analysis Kit (Biyuntian, Jiangsu, China) on a flow cytometer following the manufacturer's manual. The percentages of cells in different phases were quantified.

### 2.8. Transwell Assays

A total of 5 × 10^4^ cells in serum-free medium with 1 *μ*g/mL Mitomycin C were seeded into the upper well of a poly-carbonate transwell chamber (BD Biosciences, USA) plated in a 24-well plate. After incubation for 24 hours, cells on the upper surface of the well were scraped off with a cotton swab, and cells on the lower surface were fixed, stained, and counted.

### 2.9. Western Blotting

Proteins were retrieved from cells and equal quantities of proteins were separated by 12% sodium dodecyl sulfate-polyacrylamide gel electrophoresis, transferred to nitrocellulose membrane (Millipore, Bedford, MA, USA), and blocked with 5% bovine serum albumin. Then the membrane was incubated with MYC (Abcam, Hong Kong, China) or *β*-actin (Abcam) specific primary antibodies. After being washed by TBST for three times, the membrane was incubated with goat anti-rabbit or anti-mouse secondary antibody (Abcam) and visualized with enhanced chemiluminescence.

### 2.10. Statistical Analyses

Mann–Whitney *U* test or Wilcoxon signed-rank test was used to compare serum and tissues PVT1 expression levels between different groups as indicated. Receiver operating characteristic (ROC) curve analyses were used to calculate the diagnostic sensitivity and specificity of serum PVT1 for melanoma. Pearson correlation analysis was used to calculate the correlation between tissues and serum PVT1 expression levels. Other comparisons were assessed using Student's *t*-test. All statistical analyses were performed using the GraphPad Prism Software. *P* value of <0.05 was defined as statistically significant.

## 3. Results

### 3.1. PVT1 Is Upregulated in Melanoma Tissues

**Figure 1:**
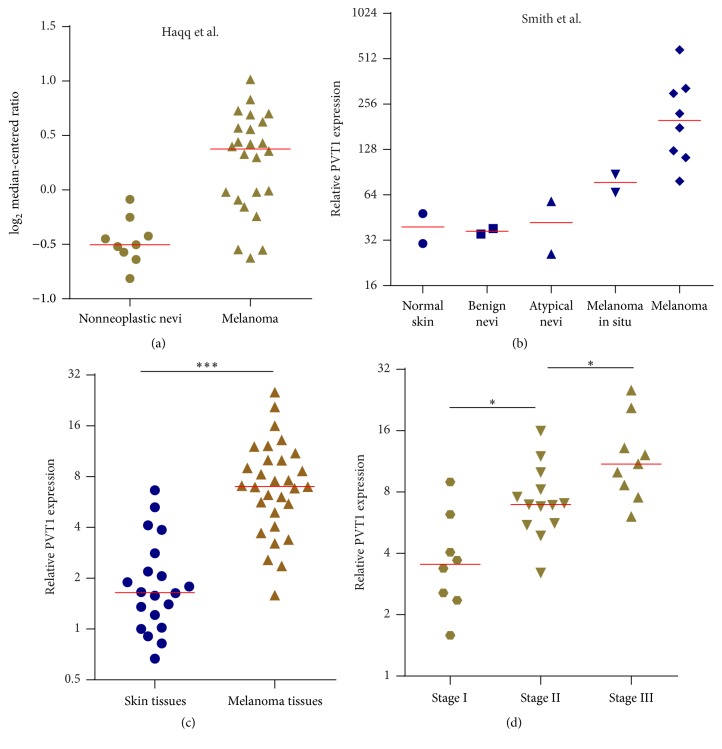
PVT1 expression levels in melanoma. (a) PVT1 expression levels in melanoma tissues and nonneoplastic nevi tissues using Oncomine expression analysis (Haqq et al., 2005). *P* < 0.001 by Mann–Whitney *U* test. (b) PVT1 expression levels in normal skin, benign and atypical nevi, melanoma in suit, and melanoma tissues using Oncomine expression analysis (Smith et al., 2005; GSE4587). (c) PVT1 expression levels in 30 malignant melanoma tissues and 20 age and gender-matched skin tissues were measured by qPCR. ^*∗∗∗*^*P* < 0.001 by Mann–Whitney *U* test. (d) PVT1 expression levels in 30 melanoma tissues with different clinical stages were measured by qPCR. ^*∗*^*P* < 0.05 by Mann–Whitney *U* test.

We investigated PVT1 expression in the publicly available melanoma data from Oncomine database. Analysis of PVT1 expression in 24 melanoma tissues and 9 nonneoplastic nevi tissues [18] revealed that PVT1 is significantly upregulated in melanoma tissues compared with nonneoplastic nevi tissues (Figure 1(a)). In another cohort representing melanomagenesis, including 2 normal skins, 2 benign nevi, 2 atypical nevi, 2 melanoma in suit, and 8 melanoma (GSE4587) [19], PVT1 is gradually increased from skins and nevi, through melanoma in suit, to melanoma (Figure 1(b)).

Because there are many alternative transcription variants of PVT1, we first detected the expression of these different PVT1 variants in melanoma tissues and A375 cells. As shown in Supplemental Figure  1 (in Supplementary Material available online at https://doi.org/10.1155/2017/7038579), PVT1 (NR_003367) is the main transcription variant in melanoma tissues and cells. We next focused our study on PVT1 (NR_003367).

To further confirm the expression pattern of PVT1 in melanoma, we collected 30 malignant melanoma tissues and 20 age and gender-matched skin tissues with melanocytic nevus and measured PVT1 expression by qPCR. As shown in Figure 1(c), PVT1 expression is significantly upregulated in melanoma tissues compared with that in control skin tissues. The 30 melanoma patients are grouped according to TNM stages, and PVT1 expression is significantly higher in later stages melanoma tissues compared with that in early stages melanoma tissues (Figure 1(d)). Collectively, publicly available PVT1 expression data and our own data all revealed the upregulated expression of PVT1 in melanoma.

### 3.2. PVT1 Is Upregulated in the Serum of Melanoma Patients and Could Be Used as a Novel Diagnostic Biomarker for Melanoma

**Figure 2:**
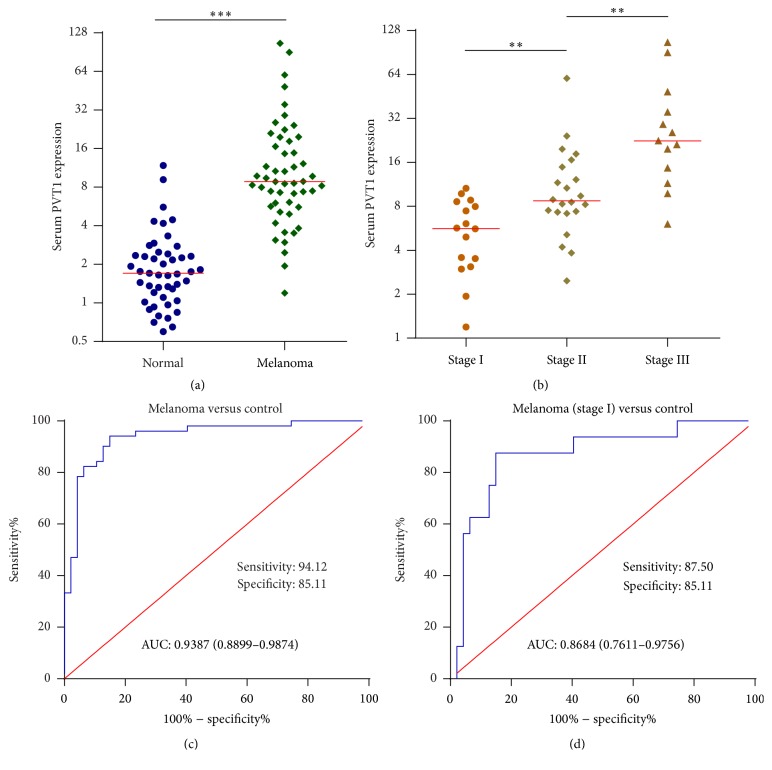
Serum PVT1 expression levels in melanoma patients and its diagnostic values for melanoma. (a) Serum PVT1 expression levels in 51 melanoma patients and 47 nonmelanoma controls with melanocytic nevus were measured by qPCR. ^*∗∗∗*^*P* < 0.001 by Mann–Whitney *U* test. (b) Serum PVT1 expression levels in 51 melanoma patients with different clinical stages were measured by qPCR. ^*∗∗*^*P* < 0.01 by Mann–Whitney *U* test. (c) ROC curve analysis of serum PVT1 levels for discrimination between melanoma patients and nonmelanoma controls with melanocytic nevus (AUC: 0.9387 (95% CI: 0.8899–0.9874), sensitivity: 94.12%, specificity: 85.11%). (d) ROC curve analysis of serum PVT1 levels for discrimination between stage I melanoma patients and nonmelanoma controls with melanocytic nevus (AUC: 0.8684 (95% CI: 0.7611–0.9756), sensitivity: 87.50%, and specificity: 85.11%).

Recently, several reports have shown that some cancer tissues highly expressed lncRNAs are detectable in serum and could be used as noninvasive biomarkers for early diagnosis of cancers [20, 21]. To investigate whether melanoma tissues highly expressed PVT1 could be used as a noninvasive biomarker for melanoma, we collected serum from 51 melanoma patients before surgery and 47 age and gender-matched nonmelanoma controls with melanocytic nevus. PVT1 expression in the serum of these patients was quantified by qPCR. As shown in Figure 2(a), serum PVT1 expression is significantly upregulated in melanoma patients compared with that in nonmelanoma controls. Moreover, serum PVT1 expression is significantly higher in later stages melanoma patients compared with that in early stages melanoma patients (Figure 2(b)).

To investigate whether serum PVT1 could be used as a biomarker for melanoma early diagnosis, ROC curve analyses were carried out. ROC curve shows accurate discrimination between melanoma patients and controls, with an area under the ROC curve (AUC) of 0.9387 (95% CI: 0.8899–0.9874), a sensitivity of 94.12%, and a specificity of 85.11% (Figure 2(c)). Furthermore, ROC curve showed good diagnostic sensitivity and specificity for stage I melanoma patients (AUC: 0.8684; 95% CI: 0.7611–0.9756; sensitivity: 87.50%; specificity: 85.11%) (Figure 2(d)). Collectively, these results showed that serum PVT1 could be used as a novel biomarker for melanoma early diagnosis.

### 3.3. Serum PVT1 Expression Could Be Used for Monitoring Melanoma Dynamics

**Figure 3:**
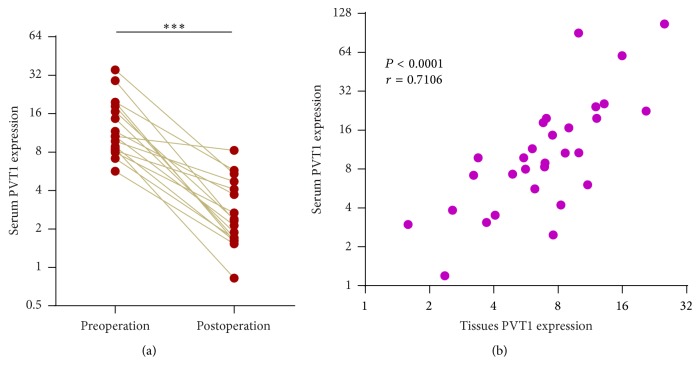
Serum PVT1 expression levels indicate melanoma dynamics. (a) Serum PVT1 expression levels in 17 pairs of preoperational and postoperational melanoma patients were measured by qPCR. ^*∗∗∗*^*P* < 0.001 by Wilcoxon signed-rank test. (b) Serum PVT1 expression levels were significantly positively correlated with melanoma tissues PVT1 expression levels. *r* = 0.7106, *P* < 0.0001 by Pearson correlation analysis.

To confirm the clinical values of serum PVT1 in monitoring melanoma dynamics, we measured serum PVT1 expression in 17 melanoma patients from both preoperational and postoperational blood. All the 17 patients received radical resection. The results showed that serum PVT1 expression is significantly reduced in postoperational melanoma patients (Figure 3(a)). To further verify whether serum PVT1 is derived from melanoma tissues, we calculated the association between PVT1 expression in melanoma tissues and melanoma patients' serum. As shown in Figure 3(b), a significant positive correlation was observed between tissues PVT1 expression and serum PVT1 expression. Collectively, these data showed that serum PVT1 is derived from melanoma tissues and may indicate melanoma dynamics.

### 3.4. PVT1 Enhances Melanoma Cells Proliferation, Cell Cycle Progression, and Migration

**Figure 4:**
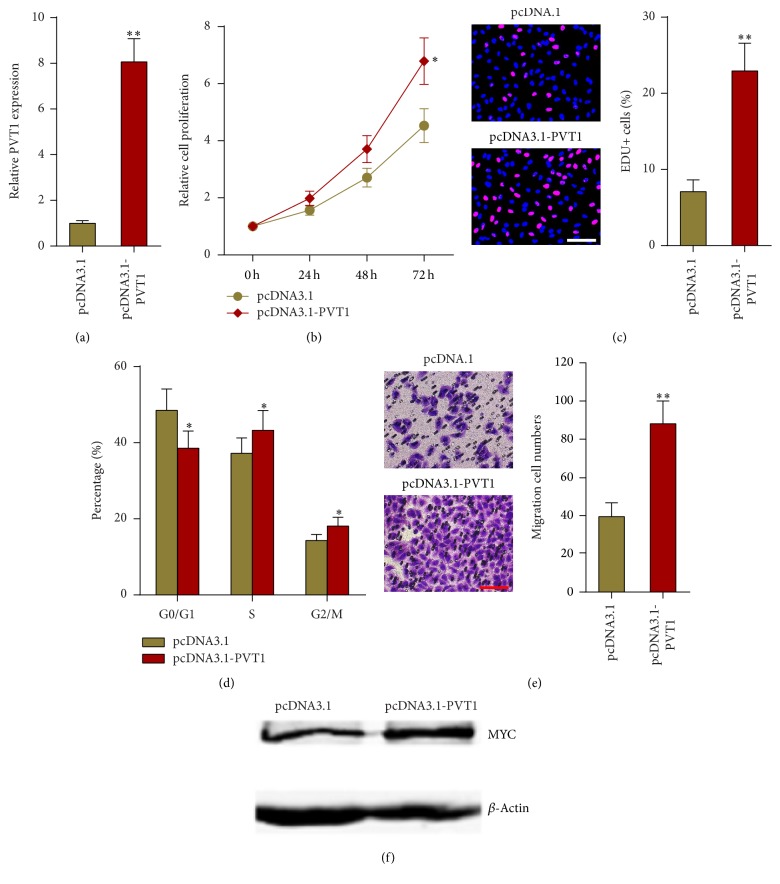
Overexpression of PVT1 enhances melanoma cells proliferation, cell cycle progression, and migration. (a) PVT1 expression levels in PVT1 stably overexpressed and control A375 cells were measured by qPCR. (b) The effect of PVT1 overexpression on A375 cells proliferation was measured by CCK-8 assays. (c) The effect of PVT1 overexpression on A375 cells proliferation was measured by EdU incorporation assays. Scale bars, 100 *μ*m. (d) The effect of PVT1 overexpression on A375 cell cycle distribution was measured by flow cytometry. (e) The effect of PVT1 overexpression on A375 cells migration was measured by transwell assays. Scale bars, 100 *μ*m. (f) Western blot analysis of MYC protein in PVT1 stably overexpressed and control A375 cells. Data are presented as mean ± SD. *n* = 3, ^*∗*^*P* < 0.05, ^*∗∗*^*P* < 0.01 by Student's *t*-test.

To explore the biological functions of PVT1 in melanoma, we stably overexpressed PVT1 in A375 cells by transfecting PVT1 expression plasmid (Figure 4(a)). Cell proliferation was evaluated using CCK-8 and EdU incorporation assays. As shown in Figures 4(b) and 4(c), both assays showed that overexpression of PVT1 significantly enhances A375 cell proliferation. Flow cytometric analyses showed that overexpression of PVT1 decreased G1/G0 phase proportion and increased S phase and G2/M phase proportion in A375 cells, suggesting its roles in the promotion of cell cycle progression (Figure 4(d)). Transwell assays showed that overexpression of PVT1 significantly promotes A375 cell migration (Figure 4(e)). PVT1 is reported to stabilize and upregulate MYC protein in breast cancer cells. MYC is a well-known oncogene in many cancers. Therefore, we detected MYC protein in PVT1 stably overexpressed and control A375 cells. As shown in Figure 4(f), overexpression of PVT1 significantly upregulates MYC protein, supporting the oncogenetic roles of PVT1 in melanoma.

### 3.5. Depletion of PVT1 Significantly Inhibits Melanoma Cells Proliferation, Cell Cycle Progression, and Migration

**Figure 5:**
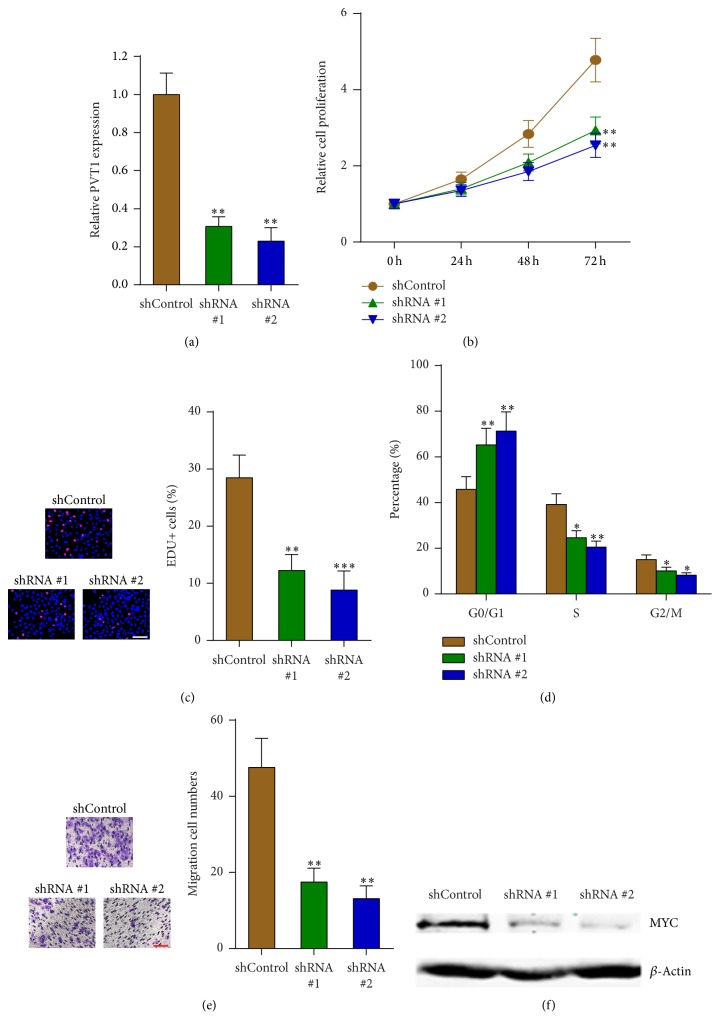
Depletion of PVT1 inhibits melanoma cells proliferation, cell cycle progression, and migration. (a) PVT1 expression levels in PVT1 stably depleted and control A375 cells were measured by qPCR. (b) The effects of PVT1 depletion on A375 cells proliferation were measured by CCK-8 assays. (c) The effects of PVT1 depletion on A375 cells proliferation were measured by EdU incorporation assays. Scale bars, 100 *μ*m. (d) The effects of PVT1 depletion on A375 cell cycle distribution were measured by flow cytometry. (e) The effects of PVT1 depletion on A375 cells migration were measured by transwell assays. Scale bars, 100 *μ*m. (f) Western blot analysis of MYC protein in PVT1 stably depleted and control A375 cells. Data are presented as mean ± SD. *n* = 3, ^*∗*^*P* < 0.05, ^*∗∗*^*P* < 0.01, and ^*∗∗∗*^*P* < 0.001 by Student's *t*-test.

To explore the therapeutic significance of targeting PVT1 in melanoma, we stably knocked down PVT1 in A375 cells using two independent PVT1 specific shRNAs (Figure 5(a)). CCK-8 and EdU incorporation assays showed that depletion of PVT1 by both shRNAs significantly inhibits A375 cell proliferation (Figures 5(b) and 5(c)). Flow cytometric analyses showed that depletion of PVT1 by both shRNAs significantly increases G1/G0 phase proportion and reduces S phase and G2/M phase proportion in A375 cells, suggesting the roles of PVT1 depletion in the inducing of cell cycle arrest (Figure 5(d)). Transwell assays showed that depletion of PVT1 by both shRNAs significantly inhibits A375 cell migration (Figure 5(e)). Western blot analysis showed that depletion of PVT1 by both shRNAs significantly decreases MYC protein in A375 cells (Figure 5(f)). Collectively, these data suggested that depletion of PVT1 significantly inhibits melanoma cell proliferation, cell cycle progression, and migration, implying that PVT1 could be a potential therapeutic target for melanoma.

## 4. Discussion

The strikingly increased occurrence rates of melanoma as well as increased prevalence of risk factors imply that melanoma will be a huge burden on society [3], which is further worsen by the refractoriness of later stage melanoma to treatment [22, 23]. Therefore, it is urgent to further understand the molecular mechanisms underlying melanoma carcinogenesis and progression and develop effective diagnostic biomarkers and therapeutic targets for melanoma.

Accumulating evidences revealed that lncRNAs exert important effects on many pathophysiological processes, such as cell cycle, cell apoptosis, cell proliferation, cell migration, drug resistance, and stem cell-like property [24–26]. Moreover, several lncRNAs have been revealed to be noninvasive serum biomarkers for various cancers [27–29]. As a highly conserved lncRNA, PVT1 has attracted great attention due to its critical and various functions in many cancers, including cervical cancer [17, 30], ovarian cancer [16], hepatocellular carcinoma [14, 31], pancreatic cancer [32], gastric cancer [33, 34], lung cancer [35, 36], and breast cancer [16]. However, the cell-type specific functions and clinical significance of PVT1 in melanoma are still unknown.

In this study, we detected PVT1 expression in publicly available melanoma data and our own cohort. All the results revealed that PVT1 is upregulated in melanoma tissues in comparation with nonneoplastic nevi tissues. Furthermore, we found that serum PVT1 level is significantly increased in melanoma patients in comparation with nonmelanoma controls with melanocytic nevus. ROC curve analyses revealed that serum PVT1 level could not only accurately discriminate melanoma patients from controls but also discriminate early stage melanoma patients from controls. Moreover, serum PVT1 level is reduced after melanoma section, and a significant positive correlation was observed between tissues PVT1 expression and serum PVT1 level. These data suggest that serum PVT1 may be a potential novel noninvasive diagnostic biomarker for melanoma and could monitor melanoma dynamics. Further studies are needed to include more samples from multiple center cohorts and from other cancers to elucidate whether serum PVT1 is specific to the diagnosis of melanoma.

PVT1 has been reported to inhibit apoptosis and enhance tumorigenicity in colorectal cancer [37], promote cell proliferation and stem cell-like property in hepatocellular carcinoma [14, 31], promote tumorigenesis in lung cancer [35], promote cell proliferation and multidrug resistance in gastric cancer [33, 38], and promote cisplatin resistance in ovarian cancer [39]. In this study, using gain-of- and loss-of-function experiments we found that overexpression of PVT1 promotes melanoma cells proliferation, cell cycle progression, and migration, while depletion of PVT1 significantly inhibits melanoma cells proliferation, cell cycle progression, and migration. These data suggest that PVT1 also has oncogenetic roles in melanoma and may be a potential therapeutic target for melanoma. To our knowledge, this is the first study to explore the functions of PVT1 in melanoma.

In conclusion, our results suggested that PVT1 is upregulated in melanoma tissues and in the serum of melanoma patients. Serum PVT1 level could be used as a sensitive and specific biomarker for melanoma early diagnosis. Functional assays revealed that PVT1 has oncogenetic roles in melanoma and could be a potential therapeutic target for melanoma.

## Supplementary Material

Supplemental Figure 1: PVT1 (NR_003367) is the main transcription variant in melanoma tissues and cells. (a) Schematic outlining the PVT1 transcription variants from UCSC (http://genome.ucsc.edu/). We named the PVT1 variants as PVT1-1, PVT1-2, PVT1-3, PVT1-4, PVT1-5 indicating the PVT1 variants shown in this figure from above to below. PVT1-4 indicts the human PVT1 revealed in NCBI (https://www.ncbi.nlm.nih.gov/), known as NR_003367. (b) The expression levels of PVT1 transcription variants in melanoma tissues and cells were measured by qPCR. The Ct values normalized by GAPDH are shown. Data are presented as mean ± SD. n = 3.
